# The effects of released pheasants on invertebrate populations in and around woodland release sites

**DOI:** 10.1002/ece3.8083

**Published:** 2021-09-14

**Authors:** Andrew Hall, Rufus A. Sage, Joah R. Madden

**Affiliations:** ^1^ Centre for Research in Animal Behaviour, Psychology University of Exeter Exeter UK; ^2^ The Game and Wildlife Conservation Trust Fordingbridge UK

**Keywords:** gamebirds, invertebrates, pheasant Phasianus colchicus, pitfall, woodland

## Abstract

The release of gamebirds for recreational shooting exerts a series of effects on the ecosystems into which they are placed. Pheasants (*Phasianus colchicus*) are omnivorous and eat invertebrates, especially when young or, if females, when breeding. Consequently, the release of large numbers of pheasants into woodland release pens may affect local invertebrate populations. Previous studies have reported mixed evidence. We conducted pitfall trapping at 13 sites (49 pens) in central England over 2 years (totaling 65 pen measures), comprising three surveys annually, immediately prior to releases in mid‐summer, 4 weeks later when most birds were still in the pens, and another 5 weeks later when most birds had dispersed. We compared traps inside and 25 m outside pens in the same wood. We considered release densities and whether the birds had prior experience of eating invertebrates. While accounting for overall seasonal declines in invertebrates trapped, we detected changes for total invertebrate biomass and total counts indicative of released pheasants causing local decreases inside pens, either directly by predation or indirectly by modifying vegetation. There were also relative decreases outside pens when the pheasants start to disperse, suggesting that the released pheasants may be affecting invertebrates in those nearby areas or that their earlier effects inside the pen, modifying vegetation or increasing invertebrate activity, increased the relative susceptibility of invertebrates there to trapping. However, these effects were not seen for specific invertebrate groups believed to be especially susceptible to pheasants. For slugs and detritivores, we detected small population increases inside pens. Across the study period, decreases for most measures were greater outside pens than inside them. We conclude that any effects pheasants have on invertebrate communities at release sites in woodlands are complex and that long‐term and taxon‐specific studies should be considered to understand the local net ecological effects of gamebird releases on invertebrates.

## Graphical abstract

The annual release of tens of millions of non‐native gamebirds for recreational shooting in the UK is considered to drive a series of ecological effects on the habitats and wildlife in and around the areas that they are introduced. One potential direct effect is that these omnivorous birds eat invertebrates and so alter their community size and composition, especially in areas where the density of released gamebirds is high. Despite the sites in our survey being examples of high density releasing where we might expect to see marked negative local effects, we found sparse consistent evidence of damage by these released birds to invertebrate communities, and for some taxa, we observed local increases in trapping rates. This suggests that released pheasants may have less direct negative effects on invertebrates than expected, and this may shift the balance of the overall net ecological effects of gamebird release.

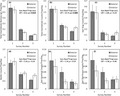

## 1 INTRODUCTION

Pheasants *Phasianus colchicus* (also red‐legged partridges *Alectoris rufa* and mallard *Anas platyrhynchos*) are released in the UK for recreational hunting. These releases comprise some tens of millions of birds annually (Madden, 2021) with the birds being managed postrelease through habitat creation, predator control, and supplementary feeding in an area influenced by shooting of over 90,000 km^2^ (PACEC2014). Their release and associated management can have a wide and mixed range of effects on the habitats and wildlife in and around the woodland release sites (Madden & Sage, 2020; Mason et al., 2020; Sage et al., 2020). The most obvious effects are seen within and in the immediate vicinity of the open‐topped pens (each occupying an area of up to several acres) into which pheasants are released when they are 6–8 weeks old, typically in July–August (Sage et al., 2020). Negative effects are more likely to occur or be more marked when release densities are high. Stocking densities of <700–1000 birds/ha are recommended by the Game and Wildlife Conservation Trust (GWCT) (Sage & Swan, 2003), and densities > 1,000 birds/ha can alter floral composition and still affect pen habitat 10 years after the pen is no longer in use (Capstick et al., 2019). Densities of birds may be especially high in and around these pens for several weeks following release, but these densities decrease as the birds disperse freely from the site and/or are predated. Therefore, the direct effects of the birds may be initially concentrated around the pen but extend further into the environment, albeit at lower intensities, as the birds disperse.

Invertebrates may provide an indicator taxon of these effects. Effects on invertebrate communities may arise indirectly because of associated changes to the habitat, either as a result of the birds themselves changing the vegetation composition (Capstick et al., 2019; Sage et al., 2005, 2009) or because the management actions in or around the sites alter vegetation composition (Hoodless & Draycott, 2008; Robertson, 1992; Short, 1994). Such effects on the habitat may increase or decrease invertebrate populations (e.g., Neumann et al., 2015; Robertson et al., 1988; Woodburn & Sage, 2005). Effects may also arise directly because pheasants in the wild may consume invertebrates, particularly when they are chicks or laying hens (Beer, 1988). Insects and other animals comprised ~5%–15% of the diet of wild‐living pheasants in the UK between July and September, based on fecal samples, with much lower proportions in the remaining months (Lachlan & Bray, 1973 in Hill & Robertson, 1988). A similar annual pattern was detected in crop/gizzard samples from the USA, but with a peak in insect and other animal consumption between June and July with levels of <5% for the rest of the year (Dalke, 1937 in Hill & Robertson, 1988). A meta‐analysis of 15 pheasant diet studies based on crop contents of 1,663 wild‐living birds collected during the spring reported that animal matter (no separation of vertebrates and invertebrates) varied from 0.9%–26.1% with a weighted average of 7.2% (Stromborg, 1979). Porter (1981) looked at 150 pheasant droppings collected from a site with pheasants and a high density of butterflies including marsh fritillary *Eurodryas aurinia* which revealed that only two samples contained caterpillar remains.

Because of the appearance of invertebrates in the diet of pheasants at certain times of year, several studies have investigated whether their releases deplete local populations of invertebrates directly, especially close to release sites where high densities of birds occur. The majority of pheasants living in the UK however are not wild‐born, but rather have been bred and reared in captivity for 6–8 weeks before being released. Therefore, we may expect that these birds are not the very young individuals, whose diet is comprised almost exclusively of invertebrates (Warner, 1979), or the laying females, which commonly eat invertebrates. Instead, the natural diet of released nonbreeding birds of 8 weeks or older is predominantly seeds and plant material (Dalke, 1937 in Hill & Robertson, 1988). Pheasant numbers are highest when birds are released and then decline steadily during the autumn preshooting and winter shooting periods (Blackburn & Gaston, 2021). Released pheasants are routinely fed throughout, so while any consumption of wild foods by these birds will normally be additional or incidental, invertebrates have been recorded as part of their diet (Doxon & Carroll, 2010; Hoodless et al., 2001). An estimated 39–57 million pheasants are released each year into UK woodlands (Madden, 2021), and consequently, even low individual levels of direct predation could affect invertebrate populations. Corke (1989) reported a negative correlation between UK 10 km^2^ tetrads where pheasants were reported (according to the BTO bird atlas) and a suite of butterfly species which he suggested could have been caused directly. However, Warren (1989) described how the size, timings, and behavior of these butterfly larvae meant that they were at a low risk of predation and that Corke's correlations were probably not causal. Clarke and Robertson (1993) conducted an experimental predation study but found no relationships with distance to pen. Clarke and Robertson (1993) also surveyed 50 woods in central southern England with historical records of fritillary colonies and found the same patterns of declines in woods with and without pheasant releasing. Pressland (2009) studied 17 matched woodland pairs (with and without pheasant releasing) in SW England. There was no detectable difference in insect numbers in wood‐edge plots with or without releasing and before or after releasing, and between any plot type after release. There were fewer insects overall caught in grass fields outside of the releasing woods compared to other woods before releasing occurred (May/June sampling), suggesting some possible chronic effects of releases. Neumann et al. (2015) looked at ground‐active invertebrates during the spring of 2 years and the autumn of 1 year at 37 woodland sites in southern England where pheasants had been released at high densities, comparing invertebrate communities within release pens and at control woodland sites. They found no difference in overall invertebrate abundance between areas inside and outside the pens. Carabid and Staphylinid species richness was also the same. However, in autumn, the release pens had fewer large woodland carabid beetles and more beetles that were characteristic of arable fields and grasslands. They suggested that release pens commonly have reduced shade due to tree canopy management. There were also more detritivores such as snails in the release pens that released more than 1,000 birds per ha.

Any link between numbers of released pheasants and changes in invertebrate populations would be strengthened by a better understanding of mechanisms underlying such links. If changes in invertebrate numbers were greater in areas where released birds were better able to catch invertebrate prey, then we might conclude that it was the direct predation by birds that was causing declines. Pheasants reared under more naturalistic (Enhanced) conditions including access to live invertebrate prey were better able to catch novel invertebrate prey and exhibited a greater dietary diversity after release (Whiteside et al., 2015). A comparison of differences in invertebrate populations between areas where these Enhanced birds and those normally reared were released might indicate whether it was the direct predation of invertebrates that caused differences in invertebrate populations or, in the absence of differences between sites, we might conclude that invertebrate populations were affected by nonpredatory effects of the released birds such as physical disturbance or changes in plant communities.

The influence of releases gamebirds on invertebrate populations would be more compelling if patterns of effects on invertebrate populations were consistent across years when release patterns remained constant. Invertebrate populations are highly susceptible to climatic conditions (Rae et al., 2006). Differences in climatic conditions between years might also distort the relationships between pheasants and the invertebrate communities. An example would be hotter and drier years resulting in increased total invertebrate biomass (Morecroft et al., 2002), possibly reducing the proportional effects of pheasant predation. There may also be carryover effects by which the presence of pheasants in 1 year caused changes in invertebrate abundance in subsequent years (Pressland, 2009). Therefore, in this study, we compared invertebrate abundance within and outside a set of 49 release pens in the West Midlands, UK, immediately prior to release, 4 weeks after release when the birds were typically still within the pen, and 9 weeks after release when birds had largely dispersed out of the pen into the surrounding landscape. We explored: Whether any effects were more likely to be driven by the density at which pheasants were released (as per Neumann et al., 2015), with the prediction that effects would be greater at higher density; whether effects differed depending on the rearing history of the birds, specifically whether those Enhanced birds reared with a more natural diet exerted greater effects on invertebrate communities; and whether these effects were consistent across 2 years when climatic conditions or a prior history of occupancy at the pen was known. We made no predictions as to which taxonomic groups would be especially affected, but rather consider this to be an exploratory study in which we initially looked at overall invertebrate populations and then refined our analyses, focusing on some common invertebrate groupings.

## 2 METHODS

### 2.1 Study area

We monitored 49 pheasant release pens in woods across 12 UK sites in Herefordshire and Worcestershire and one additional site in Leicestershire in 2016 and 2017. Sixteen pens were surveyed in both years, resulting in a total of 65 pen surveys. These pens contained a total of 59,190 pheasants, with a mean of 910 (±1SE = 210) in each. The areas surrounding the release woods were predominantly arable or pastoral fields. The mean pen area was 3,716 m^2^ (±1SE = 654 m^2^) (ESM Table 1) Stocking density of the release pens, whose area we measured from GPS points and whose bird numbers were provided by the shoot manager, ranged from 220 to 7310 birds/ha, with a mean of 2,600 birds/ha. Some of the densities that we recorded were markedly higher than those reported in other studies (Capstick et al., 2019; Neumann et al., 2015; Sage et al., 2005) and may be a consequence of using unusually small pens, each containing relatively few birds. These pens were stocked with various mixes of Enhanced birds and those that had been reared by the same breeder but using traditional methods (ESM Table 1). Briefly, traditionally reared pheasants aged 1 day old were placed in either small or large rearing setups of 350 or 800 birds respectively and confined to sheds (2.5 × 2.5 m small, 3.6 × 3.6 m large) before being given access to larger fenced and netted exterior runs of 70 m^2^ for small runs and 170 m^2^ for large runs when 2–3 weeks old (depending on the weather). Age‐appropriate food and water were continuously available *ad lib*. Pheasants were reared until 6–7 weeks old before release. Enhanced birds were reared in these same conditions but were given access to elevated perches from 1 week old and a diet of age‐appropriate commercial feed pellets supplemented with 1% live mealworms and 5% mixed bird seed. For full details of the Enhanced rearing methodology, see Whiteside et al. (2015), Whiteside et al. (2016). The weather was different across the 2 years of our study. 2017 was wetter and slightly cooler over the actual survey period (ESM Table 2).

### 2.2 Study design

**FIGURE 1:**
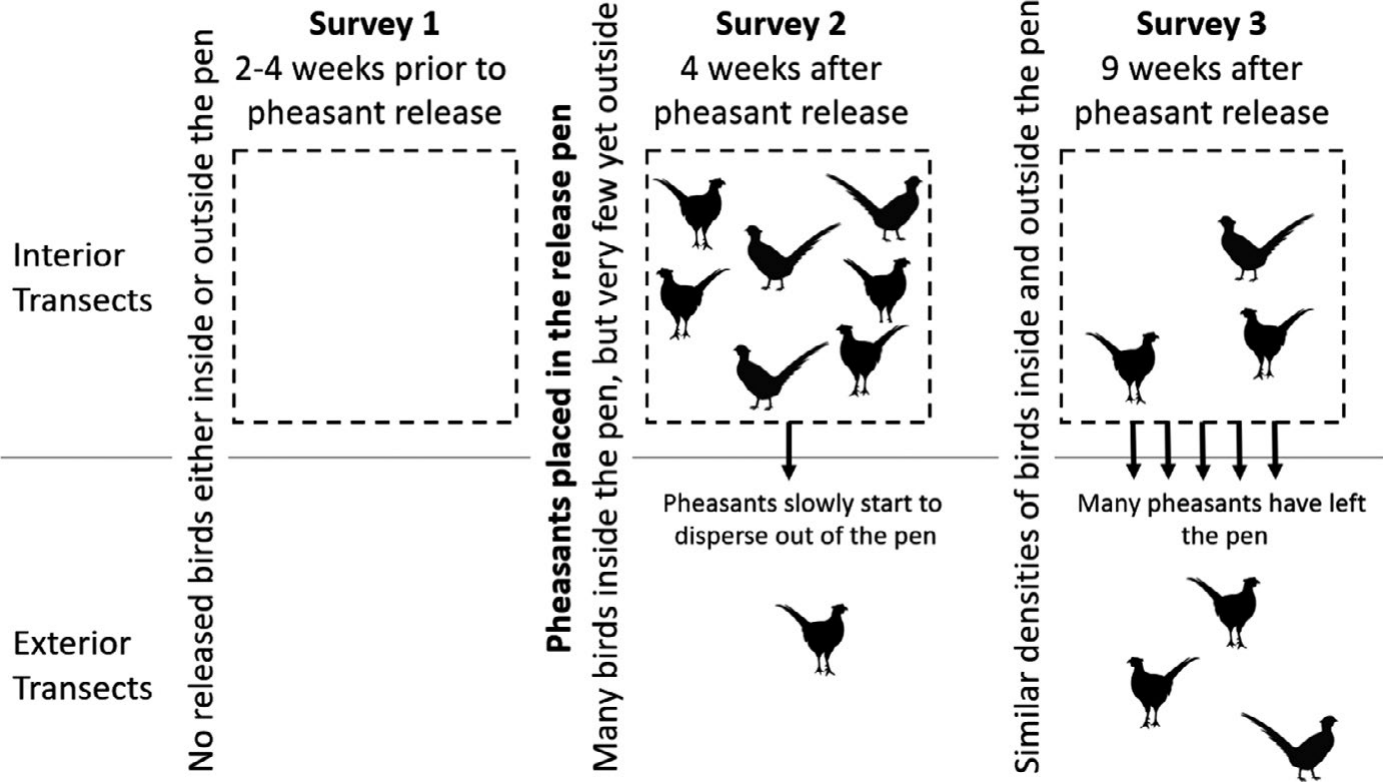
Summary of the study design showing the introduction and gradual dispersal of pheasants at release pens where transects were conducted

In order to determine the effects of released pheasants on invertebrate populations which may fluctuate due to other environmental or life‐history reasons, it is necessary to compare sites where released gamebirds are present with controls where they are absent. In the UK, where game releases are widespread and longstanding, finding sites that match the environment and faunal and floral composition of release pens but where gamebirds do not occur is difficult. An alternative is to build exclosures near to release sites. However, any exclosure that we can conceive which kept out gamebirds would also keep out a wide range of other wildlife (deer, corvids, badgers, etc.) which may have their own effects on invertebrate populations, making it hard to conclude that any effects were due to the gamebirds. Instead, we made use of the release pens and the actions of the gamekeepers at our study sites to compare transects inside the pens with transects close to but outside the pens (Figure 1). At each release pen, we conducted three surveys using pitfall trapping to sample invertebrate populations from inside (interior) and 25 m outside (exterior) of release pens. Survey 1 was conducted 2–4 weeks prior to pheasant release between 1 July and 19 August 2016 in Year 1 and 5 June and 14 August 2017 in Year 2. This allowed us to determine baseline differences in invertebrate populations between interior and exterior transects prior to pheasant releases. At this time, released pheasants were absent from both transects and we expected there to be no differences in invertebrate populations between transects due to the direct effects of released pheasants (although persistent changes to soils or vegetation from previous releases may drive chronic differences). Survey 2 was conducted 4 weeks after pheasants had been released into the pens between 12 August and 30 September 2016 and 26 July and 30 September 2017, and during which time the pheasants typically remained entirely within the pen (Beardsworth et al., 2021). This allowed us to explore the relationship between interior and exterior invertebrate populations compared to the baseline established in Survey 1. With a high density of pheasants around the interior transects but a low density around the exterior transects, differences at this point, while controlling for any underlying differences between transects at a site revealed by Survey 1, would present conditions where we could detect effects due to the presence of released pheasants. Survey 3 took place 9 weeks after the initial pheasant release between 16 September and 29 October in Year 1 and 6 August and 4 November in Year 2, by which time pheasants had typically dispersed out from the pen into the surrounding landscape. At this point, we can assume a similar density of pheasants around both transects. If the birds were having direct effects on the invertebrates, then we might expect that there would be steeper changes in samples for exterior transects which have more recently hosted pheasants compared to the interior transects that could have been affected by the presence of pheasants during Survey 2. Due to logistical issues, we were unable to conduct Survey 3 at six release pens in 2016. We collected all three surveys from all pens in 2017. This approach mimics that used by Neumann et al. (2015) in 1 year of their study (their “pen‐scale” comparison) which they contrasted with a “wood‐scale” comparison which contrasted with woods where no releases occurred. They found that the differences were basically the same across comparison type although there appeared to be lower noise in data from pen‐scale comparisons.

Neumann et al.'s (2015) and Oliver and Beattie's (1996) methodologies formed the basis for our invertebrate sampling, with surface activity of invertebrates assessed using transects of pitfall traps. A transect consisting of five interior traps, all within the pen, passed through the central point of each pen while an exterior transect of five traps was placed 25 m outside of the release pen parallel to the pen fence. In the first year (2016), we were concerned that local wildlife, particularly badgers, might disturb pitfall traps outside the pen, so we added an extra trap to each exterior transect. Subsequently, this did not generally occur in that year, so we only placed five traps on exterior transects in the second year (2017). Due to the small size of many of the release, pens only 5 m spaces were left between traps instead of 20 m recommended by studies such as Woodcock (2005). This could have potentially resulted in over‐trapping of local invertebrate populations and trapping itself decreasing invertebrate abundances (Ward et al., 2001). However, as this 5 m distance was standardized between interior and exterior transects, any negative consequences should have been equal across locations. The pens were also permeable to movements of invertebrates either through the gaps in the mesh fences, by flying over them or burrowing under them. As many of the woodlands within which the release pens were located were also relatively small, with the smallest at ~2,700 m^2^, the exterior transects were placed 25 m from the release pens to ensure that the same woodland was surveyed. Although this close proximity increased the likelihood that the exterior transects would be directly affected by the relatively nearby release pens, it also minimized potential variation in additional variables between interior and exterior transects (e.g., soil moisture, flora composition, topography, etc.). All traps that were disturbed or destroyed by wildlife between surveys were replaced within a meter of the original site for subsequent surveys to ensure consistency.

Pitfall traps consisted of buried plastic cups (200 ml) with the lip level with the soil. Covers were placed 3–5 cm above each pitfall trap to prevent rainfall flooding samples and to deter other animals from scavenging each trap's contents. Each trap was filled to one‐third with a liquid comprising 89.05% water, 10% ethylene glycol, and 0.5% Morrisons' own‐brand washing liquid. The traps were not baited. The traps were typically left for 7 days before collection. All trap contents from a single transect were pooled together. Samples were washed and sieved after collection to separate invertebrates from detritus and additional unwanted organic matter. The number of traps that survived each survey varied because of damage by wildlife, and some transects had to be collected on either 6 or 8 days after being placed, rather than the standard seven, for logistical reasons. Therefore, we standardized each invertebrate measure by correcting for trapping effort per transect, using invertebrate measure divided by trap number divided by number of operational days for our analyses.

We condensed our catch data from the pitfall traps into six invertebrate measures. We used the total biomass and total count of all individuals captured as a crude indicator of the entire invertebrate population. We then used counts for four common taxonomic groupings to allow us to investigate whether pheasants had differential impacts on particular invertebrate populations. These groupings were primarily chosen because previous research (Neumann et al., 2015) showed that pheasant releases reduced the abundance of larger carabids and increased the abundance of spiders and detritivores. Additionally, little if any research has been previously carried out on the effect of pheasants on slug populations, so slugs were also specifically investigated to expand the current knowledge base. Slugs are of particular interest as they are a major farmland pest (Frank, 1998; Martin, 1991), and as pheasants are largely released in and around arable land, any effect that pheasants might have on slugs could be directly beneficial or detrimental to farming. As such, the populations that were separately counted were as follows: Slugs (*Veronicelloidea*), Beetles (*Coleoptera*), Arachnids consisting of spiders and harvestmen (Arachnida), and Detritivores consisting of woodlice and millipedes (*Oniscidea* and *Diplopda*).

### 2.3 Statistical analysis

Due to our experimental design, we were specifically interested in the differences in invertebrate measures between the interior and exterior transects at a site, while accounting for how those differences may change over the season as invertebrate and gamebird numbers change. We wanted to test whether these patterns of difference and change were consistent across the 2 years of the study and whether either the density of the released birds or the proportion of them that had been reared under enhanced conditions and so were more likely to be efficient invertebrate predators, affected the patterns of difference and change. Because our sample size was relatively small with each pen being an independent sample, in order to address these specific questions we ran a series of models with different structures (Base Models, Density Models, and Enhanced Models). We had six invertebrate measures to consider, and therefore, we ran six sets of each model.

In each model, the comparisons of interest that might indicate that the released pheasants were having an effect or not were those between invertebrate measures collected from transects at the interior and exterior of the pen. During Survey 1, we expected that prior to the release of pheasants, measures from the interior and exterior transects would be the same. During Survey 2, we expected that with pheasants at high density inside the pen and absent or at low density outside the pen, measures from inside the pen would be lower than those outside the pen (controlling for any initial differences detected in Survey 1, as indicated by a steeper drop in the measure for the interior transects). During Survey 3, we expected that because pheasants had now dispersed out of the pen, differences between the interior and exterior transects may be smaller, although legacy effects may mean that measures from interior transects were still low, and we might expect a steeper drop in the measure for exterior transects (controlling for the numbers present during Survey 2) because the dispersing pheasants had encountered them for the first time. Seasonal changes in invertebrates may be responsible for coordinated changes for interior and exterior transects. If the effects of the pheasants on invertebrates differed between years, perhaps due to climatic conditions, then we expected to find a significant 3‐way interaction between year, survey number, and transect location. If this interaction was not significant, then we could assume that effects were consistent across the 2 years so we could drop the 3‐way interaction and the 2‐way interactions with trap location and survey number and just look at the 2‐way interaction between survey number and transect location as well as including the main effects. This critical comparison would reveal whether the invertebrate measure changed differently across the sampling season depending on whether the transects were inside or outside the pen, suggesting that pheasants were having an effect on invertebrates. These formed the set of Base Models.

To understand whether the density of released pheasants (measured as pheasants/m^2^ of pen at release; ESM Table 1) or the proportion of enhanced birds (measured as the relative number of enhanced and control birds released into the pen; ESM Table 1) exerted differential effects on the invertebrate measures, we included year as a random effect, rather than as the fixed effect that we had in the initial set of models, and instead considered the 3‐way interactions between the bird density, survey number, and transect location or between the percentage of enhanced birds, survey number, and transect location. If those interactions were significant, then we could conclude that density or percentage of enhanced birds had differential effects on invertebrate numbers in the way the measures changed over the trapping season on interior and exterior transects. These analyses formed the sets of density models and enhanced models.

All models were run in R version 3.6.1 (R Core Team, 2018) using the lme4 package (Bates et al., 2015). Generalized linear mixed effect models with a Gamma distribution and Log link function were used and a bobyqa optimizer used to improve convergence. Many invertebrate measures in a transect were 0. We are not aware of packages that allow the inclusion of mixed effects in zero‐inflated models. Additionally, we believe that the 0 measure did not truly represent absence of the invertebrate type of interest, but instead reflected their rarity, and thus, they were present at a lower level than we could detect. Therefore, we overcame the computational problem of the 0 score being impossible to analyze by adding 0.000001 to all of the invertebrate measure count/per trap/per day scores for our analyses of slugs, beetles, arachnids, and detritivores. This is <0.01% of any of the mean values of these dependent variables. Slug count, arachnid count, and detritivore count measures were square root transformed to reduce overdispersion. Significance levels for interactions and main effects were obtained using the *drop1* function.

Separate analyses were conducted on each of these invertebrate measures, but as the individual count variables are subdivisions of the total count, we recognize that the threshold for significance using *p* values should be reduced to account for multiple comparisons. However, as this was primarily an exploratory study, we retained the convention of significance being assumed when *p* < .05 while acknowledging that this increases the likelihood of Type 1 errors.

## 3 RESULTS

Although overall invertebrate measures of total biomass, total counts, slugs and beetles were all lower in 2017 compared to 2016, we found no significant 3‐way interactions between year, survey number, and trap location in any of our Base models (ESM Table 3). This indicates that any effects that the released pheasants had on invertebrate measures were consistent across the 2 years of our study. Therefore, we removed the 3‐way interaction and the 2‐way interactions between year and trap location and survey number. This allowed us to ask whether the differences between invertebrate measures differed between the interior and exterior transects differentially across the three survey periods, potentially indicating effects of released pheasants.

**FIGURE 2:**
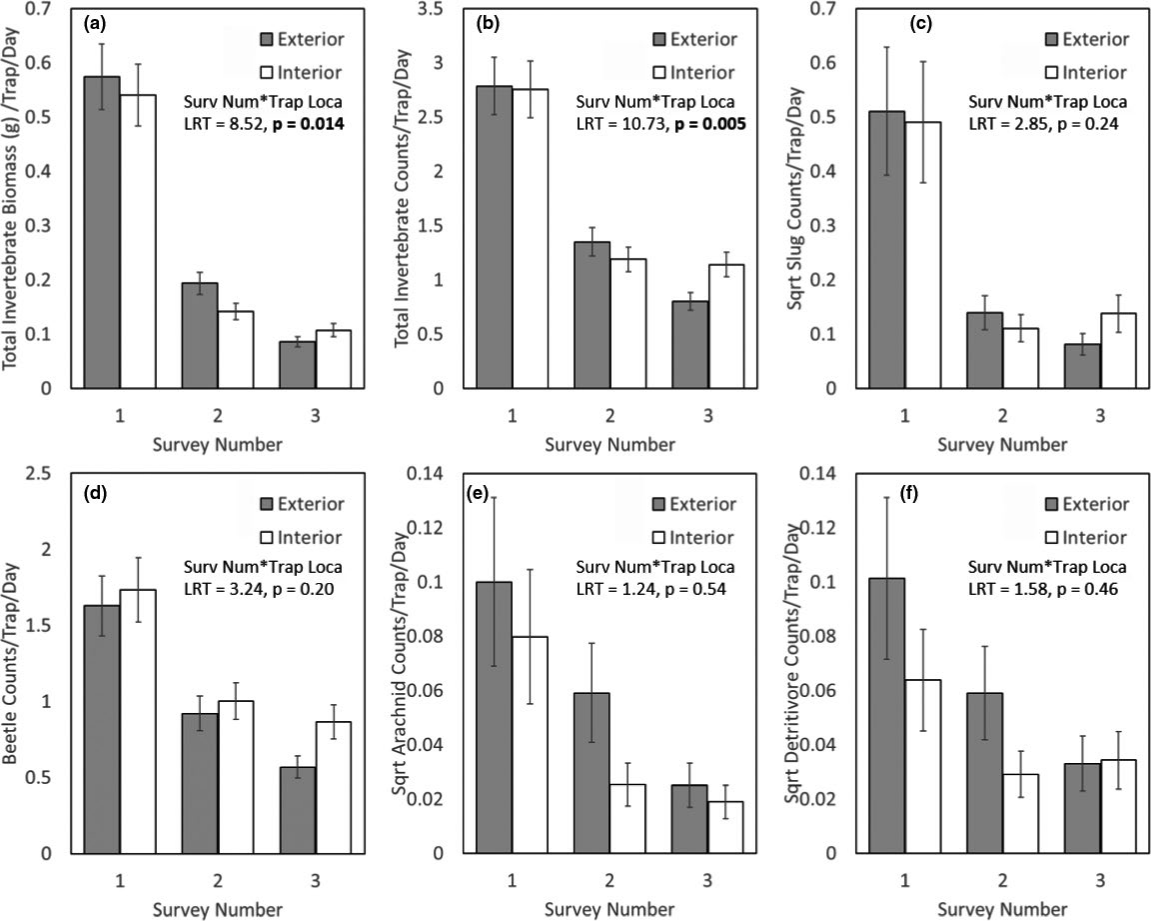
Changes in (a) total invertebrate biomass; (b) total invertebrate counts; (c) slug counts; (d) arachnid counts; (e) detritivore counts; and (f) beetle counts made inside and outside pheasant release pens. Survey number 1 = 2–4 weeks before birds were released; 2 = 4 weeks after the birds were released (and were generally still confined to the pen); 3 = 9 weeks after the birds were released (and had begun to disperse from the pen). Error bars = 1 SE. Statistics report the 2‐way interaction between Survey Number and Trap Location which might indicate that the released pheasants are having an effect on that invertebrate measure

The total invertebrate biomass and the total count measures showed separate patterns of differences between the interior and exterior transects across the survey periods, indicative of effects of released pheasants (total biomass: GLMM, trap location × survey number, LRT = 8.52, *p* = 0.014; total count: LRT = 10.73, *p* = 0.005, ESM Table 3, Figure 2). In both cases, measures were initially similar between interior and exterior transects during Survey 1 (being 6% higher on exterior compared to interior transects for total biomass and 1% higher for total counts). Measures declined markedly between Survey 1 and 2 (when pheasants were introduced to, and at high density in, the pen), and these decreases were higher for interior transects where the pheasants were present (total biomass = 74%; total counts = 57%) although there were also large decreases on exterior transects where released pheasants were absent (total biomass = 66%; total counts = 52%). These two invertebrate measures continued to decline, although at a slower rate between Survey 2 and 3 (when pheasants began to disperse from the pen) with lower decreases on interior transects (total biomass = 25%; total counts = 4%) compared to exterior transects to which pheasants were dispersing (total biomass = 55%; total counts = 41%). We did not find these differences in patterns of declines when considering the taxon‐specific measures for slugs, beetles, arachnids, or detritivores with changes across surveys being consistent between trap locations (Sqrt Slug: GLMM, trap location × survey number, LRT = 2.82, *p* = 0.24; beetle: LRT = 3.27, *p* = 0.19; sqrt arachnid: LRT = 1.08, *p* = 0.58; sqrt detritivore: LRT = 1.26, *p* = 0.53; ESM Table 3, Figure 2).

These patterns of differences in invertebrate measures between trap locations across survey periods did not differ with either density of released pheasants or the percentage of Enhanced pheasants released in a pen (GLMM, release density × trap location × survey number, all 3‐way interactions LRT < 3.92, all *p* > 0.14, full model tables in ESM Table 4; percentage of enhanced birds × trap location × survey number, all 3‐way interactions LRT < 0.4, all *p* > 0.82, full model Tables in ESM Table 5).

## 4 DISCUSSION

We detected a range of effects on invertebrates due to releasing pheasants into woodland pens. Broadly, such measures declined as the year progressed, but in general these declines for most of our measures were greatest in specific areas where pheasants had recently arrived and were expected to be at higher densities. However, these effects were not consistent across all our measures with some groupings showing increases over time; the size of the effects was small compared to overall variation between the 2 years of our survey during which time other environmental or climatic conditions may have affected invertebrate populations; we found a poor correspondence between the size of the effects and the presumed intensity of predation by the pheasants. All invertebrate measures, both from transects inside and outside the pen, were highest during Survey 1, conducted between early June and mid‐August. They were all lower during Survey 2 (conducted between late July and late September), with decreases of 42%–74% inside the pen where pheasants had been introduced, but also with simultaneous decreases of 41%–66% outside the pen where pheasants had not yet dispersed to. The invertebrate measures were again mainly lower during Survey 3 (conducted between early August and early November) compared to Survey 2, but during this period the declines were typically greater for exterior transects (38%–58%) compared to interior ones (4%–25%). Indeed, for two measures, slugs and detritivores, counts on interior transects increased (24% & 18%, respectively) over that period despite declining on exterior transects.

There were no differences between invertebrate measures inside and outside of release pens prior to release during Survey 1, indicating an absence of chronic between‐year effects on invertebrates. Pressland (2009) showed that pheasant releases can exert a chronic effect on invertebrate biomass, reducing it at the woodland scale, with evidence of lower levels of biomass prior to release. Although we did not study nonrelease woodlands, our study does show that this effect of reduced biomass from the presence of pheasants does not appear to be higher within the release pens in general. We did find that, with the exception of arachnid and detritivore counts, all 2017 invertebrate measures were significantly lower than those made in 2016 across the entire survey period, being about 2.4 times lower for Invertebrate Biomass, 3.6 times lower for Total Count and 3.6 times lower for slugs and 2.9 times lower for beetles. Naively, this might be interpreted as indicative of a chronic effect, with lower invertebrate measures in the year following a release. However, in all cases, pens had been release sites at levels similar to those occurring in our study years prior to 2016, in some cases for >15 years. Therefore, we suspect that a more likely explanation for these between‐year differences is climatic variation, with 2017 being both a drier and hotter year overall compared to 2016 but also being predominantly wetter and slightly colder over the actual survey period (Met Office, 2019). Wetter and colder weather may have reduced invertebrate movement and decreased the likelihood of them falling into the pitfall traps (Saska et al., 2013 *)*. Conversely, changes in climate may have made food less available and reduced the carrying capacity of the woodlands to support as many invertebrates (Dempster & Pollard, 1981), reducing their overall abundance.

The largest declines for all invertebrate measures occurred between Surveys 1 and 2. This period corresponds to the introduction of pheasants into the pen. It was notable that decreases were typically higher on interior transects compared to exterior transects, with the exception of counts of Beetles which decreased more on exterior transects. This suggests that the released pheasants may be having an additional effect on invertebrate declines beyond any seasonal patterns. The most marked differences (>5%) were seen for total biomass (interior declines of 74%, exterior declines of 66%), arachnid counts (interior declines of 68%, exterior declines of 41%), and detritivore counts (interior declines of 54%, exterior declines of 42%). The absolute declines, regardless of transect location, could indicate that the release of pheasants has immediate, widespread effects on invertebrate populations in woods surrounding the pens where pheasants are released. However, pheasants typically do not leave their release pen during these first few weeks (Beardsworth et al., 2021; Hill & Robertson, 1988), so we doubt whether these absolute declines are due to direct predation by the pheasants. Instead, declines outside the pen may occur because predation on invertebrates within the pen produces vacant niches that draw invertebrates in from outside and the pen may act as a population sink, so reducing their abundance outside. Alternatively, and we believe far more likely, absolute declines in Invertebrate Measures both inside and outside the pens, reflect nonpheasant related declines in populations as the year progresses (Wolda, 1988), with Survey 1 conducted between early June and mid‐August and Survey 2 conducted 6–8 weeks later. Carabids, which constituted 92% of the total beetle count, are most active in late summer, with some species being three times as active then than in winter (Cartellieri & Lövei, 2003), and predatory arthropod populations have been shown to peak in June/July (Kovanci et al., 2007). These seasonal differences would seem to match the patterns shown here.

The absolute changes in invertebrate measures between Surveys 2 and 3, when the pheasants dispersed from the release pen, were smaller and less consistent than those between Surveys 1 and 2. This may be because the invertebrate populations were naturally smaller or less active in autumn compared to summer (see above). However, when we compared changes between interior and exterior transects, decreases for all measures were greater for exterior transects (total biomass: exterior = 55%, interior = 25%; total count: exterior = 41%, interior = 4%; beetle count: exterior = 38%, interior = 14%; arachnid count: exterior = 58%, interior = 25%). For two measures, we recorded increases on interior transects over this period while exterior measures decreased (Slug count: exterior = 42%, interior = 24% increase; Detritivore count: exterior = 44%, interior = 18% increase). One potential explanation is that the trampling, disturbance, and accumulation of fecal matter inside pens may provide an attractive habitat for some particular invertebrate groups or make them more susceptible to trapping, especially once the numbers or density of pheasants in the pens has declined.

The declines in total biomass and total count measures were typically seen in areas where pheasants had recently arrived (inside the pen during Survey 2 and outside the pen in Survey 3) supporting the hypothesis that pheasants reduce invertebrate populations generally in and immediately around their release sites. However, when we considered specific taxonomic groups (arachnids, beetles, detritivores, and slugs) that we had a priori reasons to expect may be especially affected by released pheasants, we found no support for differential changes in their populations inside or outside pens during the survey period. This may be our sampling efforts were insufficient to detect small effects in subsamples of the data. The size of the overall effects on biomass and counts was small, with the declines being only 4%–9% greater inside the pen during Survey 2. This is surprising because at this point the pheasants are at their highest densities and numbers, before they have dispersed or died in large numbers, and therefore, we would expect them to exert the strongest effect if directly eating the invertebrates. We also detected relatively greater changes between exterior and interior transects during Survey 3 when pheasants had dispersed from the pen. This again supports the hypothesis that pheasants may reduce invertebrate numbers around release pens because as the birds moved out from the pens, so too did the detectable effects on some invertebrate measures. However, it is surprising that the relative (although not the absolute) larger effects seen on exterior compared to interior transects occurred later in the year for two reasons. First, as the year progresses, the proportion of invertebrate matter in pheasant diets decreases to <10% of their diet in October/November (Dalke, 1937; Lachlan & Bray, 1973, Hill & Robertson, 1988), suggesting that they may depredate insects less at this time. Second, as the birds disperse, they forage at a lower density (both because they occupy a far larger area of ground and because they die in fairly large numbers) (Madden et al., 2018), so their depredations (which are already at a lower level) are likely to be diluted. In contrast to our results supporting the hypothesis that the released pheasants caused declines in invertebrates, we also found that across all three surveys each year, there were greater total declines in five of the six invertebrate measures on exterior as opposed to interior transects. On exterior transects, decreases in total biomass, total counts slugs, beetles, and detritivores ranged from 65%–85%, whereas for interior transects, they ranged from 50%–80%. For arachnids, total exterior decreases were 75% compared to 76% on interior transects. We are not sure why areas that overall had lower numbers of pheasants present across the survey period should exhibit lower decreases in most invertebrate measures. We speculate that, if pheasants exert any effects that explain this pattern, then it is that their actions either through direct predation or damage to vegetation made invertebrates inside the release pen more susceptible to trapping, thus boosting their apparent abundance. However, we did not study invertebrate behavior or use alternative trapping methods that would allow us to better understand such a potential mechanism. Alternatively, it is possible that the changes in invertebrate measures were not caused by released pheasants, indicating that they do not have effects on invertebrate populations, matching conclusions drawn by previous studies (Clarke & Robertson, 1993; Neumann et al., 2015; Pressland, 2009; Warren, 1989).

We found little evidence that, within the range that we sampled, the density at which pheasants were stocked had any immediate differential effect on invertebrate measures, with stocking density failing to explain any of the additional variance in differences between interior and exterior transects across the survey periods as indicated by the nonsignificant 3‐way interactions involving density, trap location, and survey number. The densities of pheasant releases in this study were all relatively high, with only three of the 65 releases having stocking densities lower than the 700 birds/ha recommended by the GWCT (Sage & Swan, 2003), and only nine pen densities being lower than the 1,000 birds/ha required to avoid shifts in floral compositions and long‐term habitat degradation (Capstick et al., 2019; Sage et al., 2005). Both the mean and high outlying densities of pheasants encountered in this study are higher than reported elsewhere. Sage et al. (2005) reported a mean figure of 2,250 birds per hectare of pen in a 1988 sample of around 40 sites and a mean of 1,800 in a 2004 sample of 50 sites. Neumann et al. (2015) recorded 1,500 birds per hectare of pen at 37 sites. Our study was conducted at sites that tended to use smaller pens with correspondingly higher overall densities. Therefore, it is possible that the lack of effect of release density arose because we encountered a ceiling effect with very few pens where release densities were low enough to exert little pressure.

We found little evidence that the rearing methods used to produce the pheasants, specifically if they had prior experience of and hence greater competence at predating live invertebrates, immediately influenced any invertebrate measures with the percentage of enhanced birds released in a pen failing to explain any of the additional variance in differences between interior and exterior transects across the survey periods. Enhanced birds were only released during the years of our study, in 2016 and 2017; therefore, it was not possible that they could have exerted a chronic effect. We can conclude that the release of pheasants reared under enhanced conditions in which they might be expected to become more efficient predators of live invertebrate prey (Whiteside et al., 2015) did not have immediate disproportionate negative effects on the invertebrate community postrelease when compared to pheasants reared under more conventional methods.

It should be remembered that the results that we report here are deemed statistically significant only before any correction might account for the large number of analyses that we conducted on the various subsets of this dataset. Therefore, care should be taken when drawing conclusions from such a study and instead we recommend that our findings be used as a basis for future directed studies that specifically test some of the patterns that we report. We also emphasize that our sample of sites was relatively small and may not have captured the range of pens or shoot management techniques that occur more widely across UK lowland shoots. Our study pens appeared to be typically smaller and stocked at higher densities than those previously studied and therefore may be more representative of smaller amateur shoots than those used on larger, more professionally managed commercial shoots. There are also likely to be important interactions between the released birds and different pen floras and faunas in different regions of the UK. Despite these caveats, our results and the conclusions that we can draw from them contribute to the slowly growing picture of the direct effects of pheasants on the invertebrate communities within and around their release pens.

## 5 CONCLUSIONS

Pheasants undoubtedly eat invertebrate prey, as revealed by a range of dietary studies (Hill & Robertson, 1988; Porter, 1981; Stromborg, 1979), and so unnaturally large numbers of pheasants placed in release pens, even though they receive supplementary food, may be expected to reduce invertebrate populations. Our study involving 49 release pens over 2 years releasing at relatively high densities detected some evidence to support this hypothesis, but also indications that this is not a ubiquitous process, with those effects that we did detect often restricted to particular taxonomic groups and that the size of the effects was small compared to variation across different years when climatic or environmental effects may influence invertebrate population's size or activity. We did not explicitly look at effects on particular species, but this would be informative, especially if releases occur in areas where species of conservation concern are found (Callegari et al., 2014). The differential effects on particular invertebrate taxa that we detected could be due to direct predation, with pheasant targeting preferred, more available, or more conspicuous prey. More detailed study of pheasant foraging behavior and prey choice could help explain why some invertebrate groups may be especially vulnerable to predation. We also found that relatively higher numbers of some taxonomic groups can be found within the release pens once pheasants have dispersed into the surrounding environment. The increase in some invertebrate measures inside the pens between Surveys 2 and 3 suggests that pheasants actually make pens more attractive habitats to certain invertebrate groupings, or increases their susceptibility to trapping, through the trampling, disturbance, and accumulation of fecal matter in pens. This work indicates that the effects of released pheasants may be somewhat localized both in time and space, being detectable at a fine spatial resolution (transects separated by some tens of meters) and differentially over a four‐week period (as the birds disperse from the pen). Previous work (Sage et al., 2020) suggests that the direct effects of released gamebirds on a range of native fauna and flora are usually restricted to the areas in and around where they are released. Our study contributes to the growing body of work that reveals effects of released gamebirds on UK lowland wildlife and biodiversity, but indicates that such effects may differ depending on the biodiversity measures being used and the explicit link to predation behavior by pheasants may not be clear.

## CONFLICT OF INTEREST

None declared.

## AUTHOR CONTRIBUTIONS

**Andrew Hall:** Data curation (lead); Formal analysis (lead); Investigation (lead); Writing‐original draft (lead). **Rufus A. Sage:** Conceptualization (equal); Funding acquisition (equal); Project administration (supporting); Supervision (equal); Writing‐review & editing (equal). **Joah R. Madden:** Conceptualization (equal); Formal analysis (supporting); Funding acquisition (equal); Project administration (lead); Supervision (equal); Writing‐review & editing (equal).

## Supporting information

Appendix S1

Appendix S2

Appendix S3

Appendix S4

## Data Availability

Data and code used in this MS are provided as ESM.

## References

[ece38083-bib-0002] Bates, D. , Mächler, M. , Bolker, B. M. , & Walker, S. C. (2015). Fitting linear mixed‐effects models using lme4. Journal of Statistical Software, 67(1), 1–48. 10.18637/jss.v067.i01

[ece38083-bib-0003] Beardsworth, C. E. , Whiteside, M. A. , Capstick, L. A. , Laker, P. R. , Langley, E. J. , Nathan, R. , Orchan, Y. , Toledo, S. , van Horik, J. O. , & Madden, J. R. (2021). Spatial cognitive ability is associated with transitory movement speed but not straightness during the early stages of exploration. Royal Society Open Science, 8(3), 201758. 10.1098/rsos.201758 33959338 PMC8074888

[ece38083-bib-0004] Beer, J. V. (1988). Nutrient requirements of gamebirds. In D. J. A. Cole , & W. Haresign (Eds.), Recent advances in animal nutrition. Butterworths.

[ece38083-bib-0005] Blackburn, T. M. , & Gaston, K. J. (2021). Contribution of non‐native galliforms to annual variation in biomass of British birds. Biological Invasions, 23, 1549–1562.

[ece38083-bib-0006] Callegari, S. E. , Bonham, E. , Hoodless, A. N. , Sage, R. B. , & Holloway, G. J. (2014). Impact of game bird release on the Adonis blue butterfly *Polyommatus bellargus* (Lepidoptera Lycaenidae) on chalk grassland. European Journal Wildlife Research, 60, 781–787. 10.1007/s10344-014-0847-7

[ece38083-bib-0007] Capstick, L. , Sage, R. B. , & Hoodless, A. N. (2019). Ground flora recovery in disused pheasant pens is limited and affected by pheasant release density. Biological Conservation, 231, 181–188. 10.1016/j.biocon.2018.12.020

[ece38083-bib-0008] Cartellieri, M. , & Lövei, G. L. (2003). Seasonal dynamics and reproductive phenology of ground beetles (Coleoptera, Carabidae) in fragments of native forest in the manawatu, North Island, New Zealand. New Zealand Journal of Zoology, 30, 31–42. 10.1080/03014223.2003.9518322

[ece38083-bib-0009] Clarke, S. A. , & Robertson, P. A. (1993). The relative effects of woodland management and pheasant *Phasianus colchicus* predation on the survival of pearl‐bordered and small pearl bordered fritillaries *Boloria euphrosyne* and *B. selene* in the south of England. Biological Conservation, 65, 199–203.

[ece38083-bib-0010] Corke, D. (1989). Of Pheasants and Fritillaries: Is predation by pheasants (*Phasianus colchicus*) a cause of the decline in some British butterfly species? British Journal of Entomology and Natural History, 2, 1–14.

[ece38083-bib-0046] Dalke, P. L. (1937). Food habits of adult pheasants in Michigan based on crop analysis method. Ecology, 18(2), 199–213.

[ece38083-bib-0011] Dempster, J. P. , & Pollard, E. (1981). Fluctuations in resource availability and insect populations. Oecologia, 50, 412–416. 10.1007/BF00344984 28309062

[ece38083-bib-0012] Doxon, E. D. , & Carroll, J. P. (2010). Feeding ecology of ring‐necked pheasant and northern bobwhite chicks in Conservation Reserve Program fields. The Journal of Wildlife Management, 74, 249–256. 10.2193/2008-522

[ece38083-bib-0013] Frank, T. (1998). Slug damage and numbers of the slug pests, *Arion lusitanicus* and *Deroceras reticulatum*, in oilseed rape grown beside sown wildflower strips. Agriculture, Ecosystems and Environment, 67, 67–78. 10.1016/S0167-8809(97)00108-4

[ece38083-bib-0014] Hill, D. A. , & Robertson, P. A. (1988). The pheasant: Ecology, management, and conservation. BSP Professional Books.

[ece38083-bib-0015] Hoodless, A. N. , & Draycott, R. A. H. (2008). Pheasant releasing and woodland rides. In The game conservancy trust review of 2007 (pp. 16–17). The Game Conservancy Trust.

[ece38083-bib-0016] Hoodless, A. N. , Draycott, R. A. H. , Ludiman, M. N. , & Robertson, P. A. (2001). Spring foraging behaviour and diet of released pheasants (*Phasianus colchicus*) in the United Kingdom. In M. G. Birkan , L. M. Smith , N. J. Aebischer , F. J. Purroy , & P. A. Robertson (Eds.), Proceedings of the Perdix VII international symposium on partridges, quails and pheasants; game and wildlife science (pp. 375–386). Office National de la Chasse.

[ece38083-bib-0017] Kovanci, O. B. , Kovanci, B. , & Gencer, N. S. (2007). Species composition, seasonal dynamics and numerical responses of arthropod predators in organic strawberry fields. Biocontrol Science and Technology, 17, 457–472. 10.1080/09583150701309410

[ece38083-bib-0048] Lachlan, C. , & Bray, R. P. (1973). A study of an unmanaged pheasant population at Brownsea Island, Dorset, England. In Transactions of the 10th Congress of the International Union of Game Biologists (pp. 609–617). International Union of Game Biologists.

[ece38083-bib-0049] Madden, J. R. (2021). How many gamebirds are released in the UK each year? European Journal of Wildlife Research, 67(4), 1–4. 10.1007/s10344-021-01508-z

[ece38083-bib-0018] Madden, J. R. , Hall, A. , & Whiteside, M. A. (2018). Why do many pheasants released in the UK die, and how can we best reduce their natural mortality? European Journal of Wildlife Research, 64, 40. 10.1007/s10344-018-1199-5 32214945 PMC7088407

[ece38083-bib-0019] Madden, J. R. , & Sage, R. B. (2020). Ecological consequences of gamebird releasing and management on lowland shoots in England: A Review by Rapid Evidence Assessment for Natural England and the British Association of Shooting and Conservation. Natural England Evidence Review NEER016. Natural England.

[ece38083-bib-0020] Martin, A. (1991). Molluscs as agricultural pests. Outlook on Agriculture, 20, 167–174. 10.1177/003072709102000307

[ece38083-bib-0021] Mason, L. R. , Bicknell, J. E. , Smart, J. , & Peach, W. J. (2020). The impacts of non‐native gamebird release in the UK: An updated evidence review. RSPB Research Report No. 66. RSPB Centre for Conservation Science.

[ece38083-bib-0022] Met Office (2019). Historical station data. Met Office. https://www.metoffice.gov.uk/research/climate/maps‐and‐data/historic‐station‐data

[ece38083-bib-0023] Morecroft, M. D. , Bealey, C. E. , Howells, O. , Rennie, S. , & Woiwod, I. P. (2002). Effects of drought on contrasting insect and plant species in the UK in the mid‐1990s. Global Ecology and Biogeography, 11, 7–22. 10.1046/j.1466-822X.2002.00174.x

[ece38083-bib-0024] Neumann, J. L. , Holloway, G. J. , Sage, R. B. , & Hoodless, A. N. (2015). Releasing of pheasants for shooting in the UK alters woodland invertebrate communities. Biological Conservation, 191, 50–59. 10.1016/j.biocon.2015.06.022

[ece38083-bib-0025] Oliver, I. , & Beattie, A. J. (1996). Designing a cost‐effective invertebrate survey: A test of methods for rapid assessment of biodiversity. Ecological Applications, 6, 594–607. 10.2307/2269394

[ece38083-bib-0026] Porter, K. (1981). The population dynamics of small colonies of the butterfly *Euphydryas aurinia* . PhD thesis, Oxford University.

[ece38083-bib-0027] Pressland, C. L. (2009). The impact of releasing pheasants for shooting on invertebrates in British woodlands. PhD thesis, University of Bristol.

[ece38083-bib-0047] Rae, D. A. , Armbruster, W. S. , Edwards, M. E. , & Svengård‐Barre, M. (2006). Influence of microclimate and species interactions on the composition of plant and invertebrate communities in alpine northern Norway. Acta Oecologica, 29(3), 266–282.

[ece38083-bib-0028] R Core Team (2018). R: A language and environment for statistical computing. R Foundation for Statistical Computing.

[ece38083-bib-0029] Robertson, P. A. (1992). Woodland management for pheasants. Forestry Commission Bulletin, 106, 1–17.

[ece38083-bib-0030] Robertson, P. A. , Woodburn, M. I. A. , & Hill, D. A. (1988). The effects of woodland management for pheasants on the abundance of butterflies in Dorset, England. Biological Conservation, 45, 159–167. 10.1016/0006-3207(88)90136-X

[ece38083-bib-0031] Sage, R. B. , Hoodless, A. N. , Woodburn, M. I. , Draycott, R. A. , Madden, J. R. , & Sotherton, N. W. (2020). Summary review and synthesis: Effects on habitats and wildlife of the release and management of pheasants and red‐legged partridges on UK lowland shoots. Wildlife Biology, 2020(4). 10.2981/wlb.00766

[ece38083-bib-0032] Sage, R. B. , Ludolf, C. , & Robertson, P. A. (2005). The ground flora of ancient semi‐natural woodlands in pheasant release pens in England. Biological Conservation, 122, 243–252. 10.1016/j.biocon.2004.07.014

[ece38083-bib-0033] Sage, R. B. , & Swan, M. (2003). Woodland conservation and pheasants – Guidance leaflet. The Game Conservancy Trust.

[ece38083-bib-0034] Sage, R. B. , Woodburn, M. I. A. , Draycott, R. A. H. , Hoodless, A. N. , & Clarke, S. (2009). The flora and structure of farmland hedges and hedgebanks near to pheasant release pens compared with other hedges. Biological Conservation, 142, 1362–1369. 10.1016/j.biocon.2009.01.034

[ece38083-bib-0035] Saska, P. , van der Werf, W. , Hemerik, L. , Luff, M. L. , Hatten, T. D. , & Honek, A. (2013). Temperature effects on pitfall catches of epigeal arthropods: A model and method for bias correction. Journal of Applied Ecology, 50, 181–189. 10.1111/1365-2664.12023 PMC3607414 23539634

[ece38083-bib-0036] Short, C. (1994). Implications of game management for woodland management, landscape and conservation. Centre for Rural Studies, Royal Agricultural College.

[ece38083-bib-0037] Stromborg, K. L. (1979). Pheasant food habits in spring and consumption of seed treatment pesticides. The Journal of Wildlife Management, 43, 214–219. 10.2307/3800656

[ece38083-bib-0038] Ward, D. F. , New, T. R. , & Yen, A. L. (2001). Effects of pitfall trap spacing on the abundance, richness and composition of invertebrate catches. Journal of Insect Conservation, 5, 47–53.

[ece38083-bib-0039] Warner, R. E. (1979). Use of cover by pheasant broods in east‐central Illinois. The Journal of Wildlife Management, 43, 334–346. 10.2307/3800342

[ece38083-bib-0040] Warren, M. (1989). Pheasants and fritillaries: Is there really any evidence that pheasant rearing may have caused butterfly declines? British Journal of Entomology and Natural History, 2, 169–175.

[ece38083-bib-0041] Whiteside, M. A. , Sage, R. , & Madden, J. R. (2015). Diet complexity in early life affects survival in released pheasants by altering foraging efficiency, food choice, handling skills and gut morphology. Journal of Animal Ecology, 84, 1480–1489. 10.1111/1365-2656.12401 25994283

[ece38083-bib-0042] Whiteside, M. A. , Sage, R. , & Madden, J. R. (2016). Multiple behavioural, morphological and cognitive developmental changes arise from a single alteration to early life spatial environment, resulting in fitness consequences for released pheasants. Royal Society Open Science, 3, e160008. 10.1098/rsos.160008 PMC4821277 27069666

[ece38083-bib-0043] Wolda, H. (1988). Insect seasonality: Why? Annual Review of Ecology and Systematics, 19, 1–18. 10.1146/annurev.es.19.110188.000245

[ece38083-bib-0044] Woodburn, M. , & Sage, R. (2005). Effect of pheasant releasing on edge habitats. In The game and wildlife conservation trust review of 2004 (pp. 36–37). The Game Conservancy Trust.

[ece38083-bib-0045] Woodcock, B. A. (2005). Pitfall trapping in ecological studies. In Insect sampling in forest ecosystems, Vol. 5, (pp. 37–57). Blackwell Publishing.

