# Supplementary material for: The effects of released pheasants on invertebrate populations in and around woodland release sites
Source: Ecol Evol. 2021 Sep 14;11(19):13559–69. doi: 10.1002/ece3.8083 (PMC8495776; doi:10.1002/ece3.8083)
Supplement: Supplementary file 2 — Appendix S2 [file ECE3-11-13559-s001.docx]

**ESM for: The effects of released pheasants on invertebrate populations in and around woodland release sites**

*Andrew Hall^1^, Rufus A. Sage^2^ & Joah R. Madden^1*^*

^1.^ Centre for Research in Animal Behaviour, Psychology, University of Exeter, Exeter, EX4 4QG, UK

^2.^ The Game and Wildlife Conservation Trust, **Burgate Manor, Fordingbridge, Hampshire, SP6 1EF, UK**

*** Corresponding Author:** Centre for Research in Animal Behaviour, Psychology, University of Exeter, Exeter, EX4 4QG, UK; **j.r.madden@exeter.ac.uk**

**Table Legends:**

**ESM Table 1** Numbers of pheasants released into each pen including information on the pen size and percentage of the birds that had been reared under enhanced conditions.

**ESM Table 2** Average rainfall (mm) and temperature (°C) within the UK midlands for the survey period and average temperature and total rainfall for the entire year

**ESM Table 3** Output from **A)** base GLMMs for each of the six Invertebrate Measures with Trap Location, Survey Number and Year included as main effects and all 2-way and 3-way interactions included, with Pen ID fitted as Random effects, with p values associated with the 3-way interaction of interest (Survey Number * Trap Location * Year) derived using drop1. The Intercept refers to Survey 1 from the Exterior transect in 2017; **B)** base GLMMs for each of the six Invertebrate Measures with Trap Location, Survey Number and Year included as main effects and the 2-way interaction Survey Number * Trap Location included with Pen ID fitted as Random effects, with p values associated with the 2-way interaction of interest (Survey Number * Trap Location) and the main effect of Year derived using drop1. The Intercept refers to Survey 1 from the Exterior transect.

**ESM Table 4** Output from Density GLMMs for each of the six Invertebrate Measures with Trap Location, Survey Number and Release Density included as main effects and all 2-way and 3-way interactions included, with Pen ID and Year fitted as Random effects, with p values associated with the 3-way interaction of interest (Survey Number * Trap Location * Release Density) derived using drop1.

**ESM Table 5** Output from Enhanced GLMMs for each of the six Invertebrate Measures with Trap Location, Survey Number and Percentage of Enhanced Birds included as main effects and all 2-way and 3-way interactions included, with Pen ID and Year fitted as Random effects, with p values associated with the 3-way interaction of interest (Survey Number * Trap Location * Percentage of Enhanced Birds) derived using drop1.
